# Acute Focal Dystonia as a Presentation of Uncontrolled Hyperglycemia

**DOI:** 10.7759/cureus.71191

**Published:** 2024-10-10

**Authors:** Nicole Humphries, Bhanu Gogia

**Affiliations:** 1 Neurology, Mercer University School of Medicine, Columbus, USA; 2 Neurology, Piedmont Healthcare, Columbus, USA

**Keywords:** diabetes mellitus, focal dystonia, hyperglycemia-induced involuntary movements, movement disorder, t1w basal ganglia hyperintensity

## Abstract

Hyperglycemia-induced involuntary movements (HIIM) include tremors, hemichorea-hemiballismus (HCHB), and more rarely, dystonia. Presentations may vary, but hyperintensity involving the basal ganglia area on the T1 sequence of MRI brain remains a commonality.

We report the occurrence of focal dystonia with uncontrolled hyperglycemia but no focal abnormalities on MRI. On admission, the patient’s blood glucose was 861, and she claimed to have never missed insulin dosage. A physical exam revealed no cranial nerve abnormalities and weakness in the right upper extremity with no sensory involvement. Reflexes were 1-2+ in all extremities with down-going toes. The abnormal movements were triggered by overhead abduction of the right arm. Symptoms improved after a week with blood glucose control, as well as benzodiazepines and anticholinergics.

This specific case emphasizes the occurrence of uncontrolled hyperglycemia causing movement disorders that can have normal imaging findings. Understanding the complex presentation of patients with HIIM is pivotal for effective patient diagnosis and treatment.

## Introduction

Diabetes mellitus (DM) is associated with a myriad of neurological symptoms, including peripheral neuropathy, autonomic neuropathy, and neurovascular complications. Although more commonly associated with peripheral pathologies, uncontrolled DM can have profound effects involving the central nervous system (CNS.) These often reveal hyperintensity involving the structures of the basal ganglia on MR T1-weighted imaging [1]. The mainstay of treatment involves prompt correction of hyperglycemia, usually with IV hydration and insulin. Patients are typically able to return to baseline neurologic status after the correction and proper management of their blood glucose, although the time of improvement may vary on a case-to-case basis [2].

## Case presentation

A 28-year-old female presented with a two-day history of right upper extremity involuntary movements and a four-day history of bilateral lower extremity edema. Medical history was pertinent for hypertension, type 1 DM, and recurrent admissions for diabetic ketoacidosis (DKA), although we do not have an official record for this. She stated a recent fall of unknown origin, but it did not involve the right side of her body. On admission, the patient’s blood glucose was 861. She claimed that she had never missed insulin dosage and had been on the same regimen for many years. On physical exam, the patient was alert and oriented with no cranial nerve abnormalities. The motor exam was significant for 4+/5 strength of the right biceps, 4/5 strength in the right triceps, and 2/5 strength of the right wrist extensors and interossei with no sensory involvement. Reflexes were 1-2+ with down-going toes. The abnormal movements typical for dystonia were triggered by overhead abduction of the right arm, as seen in the attached video (Video 1). The dystonic movements would continue for a few minutes.

**Video 1:** Abnormal movements elicited by overhead abduction of the right arm

**Figure 1:**
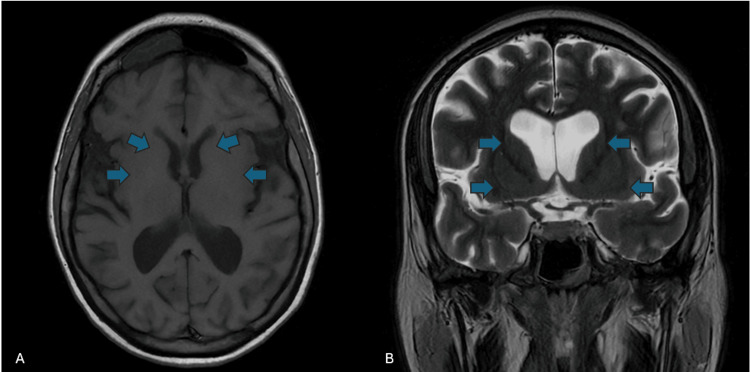
MRI brain on day one of admission (A) displaying axial T1 image with microvascular changes but no hyperintensities or focal abnormalities and (B) displaying coronal T2 images without hyperintensities or focal abnormalities

The biochemical profile is shown in Table 1 and reveals a general decrease in electrolyte levels, which increased with the improvement of hyperglycemia (Table 1). Pertinent positives included elevated hemoglobin A1C of 11.2 (normal range <6%), serum osmolality of 315 (normal range 275-295), and a mildly elevated beta-hydroxybutyrate at 1.98 (normal <0.5). Urinalysis was negative for ketones. MRI brain was obtained with and without contrast and revealed chronic microvascular and atrophic changes, but no hyperintensities or abnormalities were noted in the basal ganglia (Figure 1). MRI C-spine and MRI brachial plexus were done to look for brachial plexopathy and were unremarkable. GAD 65 was undetectable.

**Table 1:** Relevant values through the hospital stay

Date	Day 1	Day 2	Day 3	Day 4	Normal range
Glucose, random	861	235	385	171	<200 mg/dL
Sodium, Na	125	132	136		135-145 mEq/L
Potassium, K	3.4	3.5	4.1		3.5-5 mEq/L
Creatinine, Cr	0.92	0.7	0.92		0.7-1.3 mg/dL
GAD- 65			<5		<5 IU/mL

The patient was started on insulin lispro and glargine in the hospital, which was able to lower blood glucose to as low as 171. She was prescribed a one-month supply of Cogentin 1 mg twice a day (BID), Flexeril 5 mg thrice a day (TID), and Klonopin 0.5 mg BID. She was discharged on day three of hospitalization and referred to follow-up with endocrinology to achieve optimal blood glucose control and outpatient neurology two-to-four weeks after discharge.

## Discussion

With only mildly elevated beta-hydroxybutyrate, osmolality of 315, and negative urinalysis for ketones, DKA is an unlikely cause of this hyperglycemic state. The osmolality is also below the range of >320 for the diagnosis of hyperosmolar hyperglycemic state per the American Diabetes Association. The general decrease in electrolytes in this case was most likely a relative decrease due to the significant increase in blood glucose, as the values improved with the correction of hyperglycemia. One exception was seen with elevated blood glucose on day three compared to day two. This still presented with increased electrolyte levels, and it is suspected that this was secondary to subtherapeutic insulin dosing.

Neurologic damage in hyperglycemia-induced involuntary movements (HIIM) may be explained by vascular insufficiency, GABA depletion, or hyperosmolar insult [3]. Although many cases of hemichorea-hemiballismus (HCHB) have been documented, acute focal dystonia secondary to hyperglycemia is poorly discussed in the literature. Dystonia is defined by the American Association of Neurologic Surgeons as involuntary muscle contractions resulting from basal ganglia dysfunction [4]. It is a common movement disorder but is very rarely documented in congruence with hyperglycemia.

Although the exact mechanism remains unclear, MRI findings in HIIM often reveal T1 hyperintensities in the basal ganglia structures [5]. This patient’s case remains especially unique due to her rather unremarkable MRI and dystonic features. Although it did reveal mild atrophy and chronic microvascular ischemic changes that are advanced for her age, there were no hyperintensities noted in the basal ganglia, as one may expect with any form of HIIM. One other case was cited as focal dystonia presenting with torticollis and an unremarkable basal ganglia on MRI. This patient did, however, reveal a calcification in the frontal lobe [6]. Akande et al. discuss a similar presentation with unilateral upper extremity involuntary movement and a fasting blood glucose of 400 mg/dL. Although the MRI was withheld due to financial concerns, the CT showed hyperdense signals within the globus pallidi [7].

As hyperglycemia improves and the symptoms diminish, a follow-up MRI typically reveals the resolution of the abnormalities. Without MRI anomaly, resolution indications and prognostic evaluation are limited, eliciting difficulty in progression management or reoccurrence assessment.

Our patient was still experiencing symptoms at discharge, but it is common for the symptoms to improve more gradually [2]. Toxin exposure and electrolyte abnormalities may present with dystonic features, but our initial laboratory tests supported hyperglycemia as the insulting factor. Glycemic control was still the management of choice in this patient, but clonazepam and benztropine (anticholinergic) were utilized to minimize symptoms during the resolution of HIIM. The patient reported being symptom-free on telephone follow-up one month after discharge. She is in the process of finding an outpatient neurologist as she recently moved out of the state.

## Conclusions

Hyperkinetic movements associated with hyperglycemia could be due to tremor or hemiballismus-chorea, with cases often associated with signal changes in basal ganglia on MRI. Focal dystonia is not as commonly reported, and this specific case highlights the occurrence of other movement disorders associated with uncontrolled hyperglycemia that could have normal imaging findings. Understanding the complex presentation of patients with HIIM is pivotal for effective patient diagnosis and treatment.
